# The ‘Shellome’ of the Crocus Clam *Tridacna crocea* Emphasizes Essential Components of Mollusk Shell Biomineralization

**DOI:** 10.3389/fgene.2021.674539

**Published:** 2021-06-08

**Authors:** Takeshi Takeuchi, Manabu Fujie, Ryo Koyanagi, Laurent Plasseraud, Isabelle Ziegler-Devin, Nicolas Brosse, Cédric Broussard, Noriyuki Satoh, Frédéric Marin

**Affiliations:** ^1^Marine Genomics Unit, Okinawa Institute of Science and Technology Graduate University, Onna, Okinawa, Japan; ^2^DNA Sequencing Section, Okinawa Institute of Science and Technology Graduate University, Onna, Okinawa, Japan; ^3^Institut de Chimie Moléculaire de l’Université de Bourgogne, UMR CNRS 6302, Faculté des Sciences Mirande, Université de Bourgogne - Franche-Comté (UBFC), Dijon, France; ^4^LERMAB, Faculté des Sciences et Technologies - Campus Aiguillettes, Université de Lorraine, Vandoeuvre-Lès-Nancy, France; ^5^3P5 Proteomic Platform, Cochin Institute, University of Paris, INSERM U1016, CNRS UMR 8104, Paris, France; ^6^UMR CNRS 6282 Biogéosciences, Bâtiment des Sciences Gabriel, Université de Bourgogne - Franche-Comté (UBFC), Dijon, France

**Keywords:** biomineralization, transcriptome, proteome, Mollusca, Bivalvia, *Tridacna crocea*, shell formation

## Abstract

Molluscan shells are among the most fascinating research objects because of their diverse morphologies and textures. The formation of these delicate biomineralized structures is a matrix-mediated process. A question that arises is what are the essential components required to build these exoskeletons. In order to understand the molecular mechanisms of molluscan shell formation, it is crucial to identify organic macromolecules in different shells from diverse taxa. In the case of bivalves, however, taxon sampling in previous shell proteomics studies are focused predominantly on representatives of the class Pteriomorphia such as pearl oysters, edible oysters and mussels. In this study, we have characterized the shell organic matrix from the crocus clam, *Tridacna crocea*, (Heterodonta) using various biochemical techniques, including SDS-PAGE, FT-IR, monosaccharide analysis, and enzyme-linked lectin assay (ELLA). Furthermore, we have identified a number of shell matrix proteins (SMPs) using a comprehensive proteomics approach combined to RNA-seq. The biochemical studies confirmed the presence of proteins, polysaccharides, and sulfates in the *T. crocea* shell organic matrix. Proteomics analysis revealed that the majority of the *T. crocea* SMPs are novel and dissimilar to known SMPs identified from the other bivalve species. Meanwhile, the SMP repertoire of the crocus clam also includes proteins with conserved functional domains such as chitin-binding domain, VWA domain, and protease inhibitor domain. We also identified BMSP (Blue Mussel Shell Protein, originally reported from *Mytilus*), which is widely distributed among molluscan shell matrix proteins. *Tridacna* SMPs also include low-complexity regions (LCRs) that are absent in the other molluscan genomes, indicating that these genes may have evolved in specific lineage. These results highlight the diversity of the organic molecules – in particular proteins – that are essential for molluscan shell formation.

## Introduction

The shell of mollusks represents a major biological innovation that largely contributed to the great evolutionary and ecological success of the phylum throughout Phanerozoic times. As the shell offered a robust shelter against predation and an effective protection against desiccation, it allowed mollusks to conquer – from shallow epicontinental marine seas – all kinds of habitats, including deep-sea hydrothermal vents, brackish and freshwater domains or a large array of terrestrial environments: in this latter case, even the most hostile ones like deserts, caves, polar territories and high mountains were colonized and still are.

Beside complex genetic equipment involved in development, the formation of mollusk shell is a highly regulated process that requires a large set of macromolecules, i.e., proteins, polysaccharides and lipids, secreted from the dorsal mantle tissue, and which self-assemble into an organic matrix, the framework for shell mineralization. These macromolecules – in particular shell matrix proteins, defined here as SMPs - also play crucial role in nucleation and growth of calcium carbonate crystal. They are in addition considered to be key-players for controlling shell microstructures and mineralogy (Marin et al., 2007).

Our knowledge of mollusk SMP repertoires is rapidly expanding, due to the combined use of transcriptomics on calcifying mollusk mantle tissues and proteomics on shell macromolecular extracts (Joubert et al., 2010; Marie et al., 2011a; Marin et al., 2013). Accumulating data of shell matrix proteomes - referred to as ‘shellomes’ – from different species highlights extensive but unsuspected diversification of SMP repertoires, in particular in bivalves, the most studied mollusk class from a biomineralization viewpoint (Kocot et al., 2016; Marin, 2020). This diversity expresses not only from genus to genus but also between different shell microstructures of a single shell. For instance, the pearl oysters (genus *Pinctada*) synthesize nacreous aragonitic layer in the inner side of their shells and a calcitic prismatic one in the outer part. To this end, they secrete two different SMP repertoires from corresponding mantle region (Marie et al., 2012; Zhao et al., 2018). Furthermore, to render the situation more complex, it has been shown that larval shell SMP repertoires are almost entirely different from that of adult shells (Zhao et al., 2018). Beside this diversity, some common characteristics can be identified: they include the presence of shared functional domains and the abundance of low complexity regions, referred to as LCRs (Kocot et al., 2016; Marie et al., 2017). These conserved elements may be a clue to understand the evolutionary origin of shell biomineralization. In order to draw a general view of biomineralization process and its evolutionary origin, it is essential to compile a “dictionary” of SMPs that is identified from diverse range of taxa.

To date, shell matrix proteomes, ‘shellomes,’ were reported from 20 bivalve genera (Marin, 2020). Among them, half belong to Pteriomorphia sub-class, including the pearl oysters *Pinctada margaritifera*, *Pinctada maxima*, and *Pinctada fucata* (Joubert et al., 2010; Marie et al., 2012; Liu et al., 2015; Zhao et al., 2018), the Pacific cupped oyster *Crassostrea gigas* (Zhang et al., 2012; Arivalagan et al., 2017; Zhao et al., 2018), the edible mussels including *Mytilus edulis*, *Mytilus galloprovincialis*, *Mytilus californianus*, *Mytilus coruscus*, and *Perna viridis* (Marie et al., 2011a; Gao et al., 2015; Liao et al., 2015, 2019; Arivalagan et al., 2017), and the king scallop *Pecten maximus* (Arivalagan et al., 2017). In contrast, SMPs of sub-classes Palaeoheterodonta and Heterodonta (Plazzi and Passamonti, 2010) have been far less studied. To our knowledge, the shell proteomes supported by a transcriptome of 3 Palaeoheterodonta species have been analyzed in detail, corresponding to unionoid freshwater mussels: *Hyriopsis cumingii*, *Elliptio complanata*, and *Villosa lienosa* (Berland et al., 2013; Marie et al., 2017). For Heterodonta *sensu lato* (including Anomalodesmata), “shellomic” data supported by transcriptome are scarce too: they include that of the clams *Venerupis philippinarum* (Marie et al., 2011b), *Mya truncata* (Arivalagan et al., 2016), and *Laternula elliptica* (Sleight et al., 2015). Despite the increasing list of SMPs, it should be noted that our present view of biomineralization process in bivalves is extremely partial, given the size of this class (12,000 living species). Entire clades, like heterodont or protobranch bivalves, remain to be investigated.

To this end, we analyzed the shell organic components of the crocus clam, *Tridacna crocea*, a heterodont bivalve and one of the modest-sized representatives of the giant clam taxon. Giant clams, in particular *T. gigas*, are one of the most fascinating models in biomineralization research since they produce the largest and heaviest shells among mollusks. All *Tridacna* species produce thick, dense and rigid shells mostly of crossed lamellar microstructure (Taylor, 1973; Dauphin and Denis, 2000; Agbaje et al., 2017; Gannon et al., 2017). As the giant clams continue to grow their shells throughout the life, shells are precise recorders of environmental conditions such as seawater temperature (Arias-Ruiz et al., 2017; Yan et al., 2020). The longevity of giant clams allows the shells to archive long-term environmental conditions, with a resolution ranging from century to month, or even higher (Yan et al., 2020).

The crocus clam, *T. crocea*, is distributed in tropical seawaters spreading from the western Pacific (Japan, New Caledonia) to the eastern Indian Ocean (Lucas, 1988). *T. crocea* hosts photosymbiotic dinoflagellate algae (Hirose et al., 2006; Ikeda et al., 2017) and they acquire nutrition from both filter-feeding and photosynthesis via zooxanthellae, allowing their fast growth. Despite their ability to produce large amount of calcium carbonate shells, the molecular basis underlying their shell formation has never been explored. In this study, we conducted a biochemical characterization of *T. crocea* organic shell matrix and furthermore identified SMPs constituting the ‘shellomes’ of the crocus clam by using a combination of proteomics on shell extracts and transcriptomics on mantle tissue. Our results highlight essential components for shell formation in this peculiar crossed-lamellar bivalve model.

## Materials and Methods

### Sample Collection

An adult of *T. crocea* (approx. 8 cm in length) was collected at the Onna fisheries corporation, Okinawa, Japan. Soft tissues were separated from the shells and the mantle tissues were immediately used for RNA extraction (see below). The two valves were immersed in 1% NaOCl solution for 24 h (initial bleaching), mechanically cleaned to remove remaining tissues, superficial epibionts and periostracum and rinsed with deionized water (DI water, 18 MΩ). The shells were crushed into ∼2 mm fragments with a Jaw-crusher (Retsch BB200), followed by incubating in 1% NaOCl for 60 h (second bleaching). The fragments were then washed twice with DI water, dried, and powdered using a mortar grinder (Frisch Pulverisette 2). The powder (81.6 g) was sieved (pore size <200 μm) and separated into two batches. The first was subsequently decalcified, while the second was bleached for an additional 16 h in 1% NaOCl solution (third bleaching), then thoroughly washed (DI water) and air-dried at 37°C before decalcification.

### Extraction of Shell Matrices

The cleaned powder samples (second or third bleaching, approx. 40 g each) were suspended in cold water and decalcified overnight at 4°C by progressively adding (100 μL every 5 s.) cold dilute acetic acid (10% vol/vol) with an electronic burette (Titronic Universal, Schott, Mainz, Germany). The solution was centrifuged at 3,900 *g* for 30 min to separate the supernatant and the pellet. The supernatant was filtered (5 μm) on a Nalgene filtration apparatus and concentrated by ultrafiltration (Amicon stirred cell 400 mL) on a 10 kDa cutoff membrane (Millipore, ref. PLGC07610). The concentrated solution (approx. 16 mL) was dialyzed 4 days against MilliQ water with several water changes, and lyophilized to obtain the acid-soluble matrix (ASM). The pellet was resuspended in Milli-Q water, centrifuged, and the supernatant discarded. After three cycles of resuspension-centrifugation-supernatant discarding, the pellet was lyophilized, forming the acid-insoluble matrix (AIM).

### SDS-PAGE

AIM and ASM were suspended in 1× Laemmli sample buffer (Laemmli, 1970) containing β-mercaptoethanol. Samples were denatured for 5 min at 99°C, cooled on ice and briefly centrifuged. Note that for AIM only a fraction of this matrix can be dissolved in Laemmli buffer. This soluble matrix is referred to as LS-AIM (Laemmli-soluble – Acid-insoluble matrix). Then, supernatant were run on precast 4–20% gradient polyacrylamide mini-gels (Bio-Rad) in mini-Protean III system. Gels were stained with silver nitrate (Morrissey, 1981), Stains-all for putative calcium-binding proteins (Campbell et al., 1983; Marin et al., 2005) and Alcian blue, for polyanionic macromolecules/sulfated sugars (Thornton et al., 1996) at pH 1.0.

### FT-IR Spectroscopy

FT-IR spectra were acquired on all AIM extracts and on the different cleaned shell powders. In this latter case (not illustrated here), we verified that the powders were all aragonitic, with the double absorption band at 700–712 cm^–1^, the one at 857 cm^–1^, the thin low amplitude absorption band at 1,083 cm^–1^ and the main absorption band at 1,474 cm^–1^. All samples were measured using a Bruker Vector 22 FT-IR spectrometer (Bruker Optics Sarl, Marne la Vallée, France) equipped with a Specac Golden Gate Attenuated Total Reflectance (ATR) device (Specac Ltd., Orpington, United Kingdom) in the wavenumber range 4,000–500 cm^–1^ (12 scans at a spectral resolution of 4 cm^–1^). The background was recorded before each measurement. The qualitative assignment of absorption bands was performed manually by comparison with previously described spectra, carried out by our group or available in the bibliography.

### Monosaccharide Analysis

Monosaccharide quantification of AIMs after two or three bleaching steps was performed according to the HPAE-PAD technology (High Pressure Anion-Exchange - Pulsed Amperometric Detection) on an ICS-3000, Dionex system equipped with a Dionex CarboPac^TM^ PA-20 (3 mm × 150 mm) analytical column. In short, lyophilized samples were hydrolyzed in 2 M trifluoroacetic acid at 105°C for 4 h (100 μg/100 μL), and the solution was neutralized with sodium hydroxide. Hydrolytic conditions deacetylate *N*-acetyl-glucosamine and *N*-acetyl-galactosamine, which are subsequently analyzed as glucosamine and galactosamine, respectively. Filtered samples (20 μL) were eluted at 0.4 mL/min (35°C) using the following sodium hydroxide gradient: pure water 99.2%/250 mM NaOH 0.8%: 0 ∼ 20 min; pure water 75%/250 mM NaOH 20%/NaOAc (1M)- NaOH (20 mM) 5%: 20 ∼ 37 min; pure water 40%/250 mM NaOH 20%/NaOAc (1M)-NaOH (20 mM) 40%: 37 ∼ 41 min. Each elution was followed by a wash and subsequent equilibration time. External sugar and uronic acids standards were used for calibration (7 points per curve): fucose, glucose, xylose, galactose, mannose, rhamnose, arabinose, glucosamine, galactosamine, galacturonic acid, and glucuronic acid (all provided by Sigma-Aldrich).

### Enzyme-Linked Lectin Assay (ELLA)

Enzyme Linked Lectin Assay (ELLA) was conducted as described previously (Immel et al., 2016; Takeuchi et al., 2018) on ASM fractions only. Briefly, 96-well plates (MaxiSorp, Nunc/Thermo Scientific, Nunc A/S, Roskilde, Denmark) were coated with ASM (50 ng/well) and incubated for 90 min at 37°C. They were washed three times with a solution of TBS/Tween-20 (0.5 mL Tween 20 per L) spread using a manual microplate 8-channel washer (Nunc Immuno Wash), and subsequently blocked with Carbo-free blocking solution (Vector Laboratories, ref. SP-5040) for 60 min at 37°C. Three sets of 7 biotinylated lectins were used (Vector Laboratories, Peterborough, United Kingdom, ref. BK-1000, BK-2000, and BK-3000). Lectins were applied to the wells (dilution to 10 μg/mL) and incubated for 90 min at 37°C. Unbound lectins were washed five times with TBS/Tween-20. Then, a solution containing alkaline phosphatase-conjugated avidin (Avidin-AP, Sigma A7294, St. Louis, MO, United States) diluted 70,000 times was added (100 μL per well) and incubated for 90 min at 37°C. Microplates were washed again and incubated with ELISA substrate solution (10% vol/vol diethanolamine in Milli-Q water, pH 9.8) containing phosphatase substrate [0.5 mg/mL, 4-nitrophenyl phosphate disodium salt hexahydrate (pNPP) tablet, Sigma, ref. UN3500-A] at 37°C. They were read every 15 min at 405 nm using a Bio-Rad Model 680 micro-plate reader. Results were normalized and converted to percentage of reactivity by subtracting the background (negative control comprising ASM without lectin but with Avidin-AP) from all values and by considering the highest response as 100%. The test was repeated three times. For detailed information on the saccharidic target binding-sites of each lectin, see one of our previous references (Immel et al., 2016).

### Mantle Transcriptome

Total RNA of *T. crocea* was extracted from an adult mantle tissue using Trizol reagent (Invitrogen) and purified using an RNeasy micro kit with DNase (QIAGEN). RNA-seq libraries were prepared using a TruSeq RNA sample Prep Kit v2 (Illumina) following the manufacture’s protocol and sequenced with the Illumina GAIIx platform. Raw sequences were quality filtered and trimmed with Trimmomatic 0.36 (Bolger et al., 2014). Reads were then assembled using Trinity version r20140413p1 (Grabherr et al., 2011).

### Shell Proteome

Proteomic analyses were conducted on the bulk ASM and AIM matrices (obtained after two and three bleaching treatments) after an in-gel digestion with trypsin, as previously published (Immel et al., 2016). For MS and MS/MS ORBITRAP, analyses were performed using an Ultimate 3000 Rapid Separation Liquid Chromatographic (RSLC) system (Thermo Fisher Scientific) online with a hybrid LTQ-Orbitrap-Velos mass spectrometer (Thermo Fisher Scientific). All technical details are provided in earlier work (Immel et al., 2016). Database searches were carried out using Mascot version 2.4 and 2.5 (Matrix Science, London, United Kingdom) on the transcriptome of *T. crocea*. The false discovery rate was set to 0.05. Proteins supported by more than one peptide sequence were identified as shell matrix proteins (SMPs) in this study. The most significant results are presented in this study.

### Characterization of Protein Sequences

Protein sequences identified through proteomic analysis were analyzed using the InterProscan (ver. 5.14-53.0) platform (Jones et al., 2014) in order to search for functional domains. Signal peptide prediction was conducted with SignalP 4.0 (Petersen et al., 2011). Transmembrane domains were assessed with TMHMM version 2.0 (Krogh et al., 2001). Protein sequences were also Blastp-searched against nr or UniProt databases. Theoretical molecular weights and isoelectric points of the proteins were calculated based on their amino acid sequences using IPC 1.0 (Kozlowski, 2016). Phosphorylation sites in protein sequences were predicted using NetPhos 3.1 (Blom et al., 1999). In order to identify LCRs in protein sequences, we searched polypeptide sequences where majority of the amino acids were composed of one or two amino acids. LCRs were defined based on the following criteria; (i) single amino acid occupies ≥50% of the 10 amino acid sequence window in more than 14 successive polypeptides or (ii) the most abundant amino acid occupies ≥40% and the second abundant amino acid occupies ≥20% of the 10 amino acid sequence window in more than 19 successive polypeptides.

## Results

### SDS-PAGE

**FIGURE 1:**
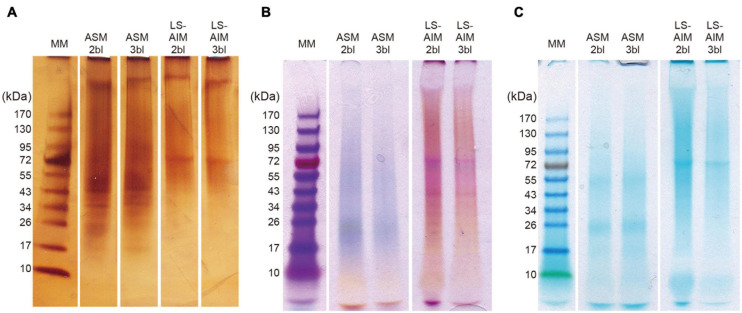
SDS-PAGE of shell organic matrices. **(A)** Silver staining, **(B)** stains-all staining, **(C)** alcian blue staining. Ten microliter of sample solution was loaded in each lane for silver staining and 15 μl for Stains-all staining and alcian blue staining. ASM, acid-soluble matrix. LS-AIM, Laemmli-soluble acid-insoluble matrix. 2bl, second bleaching. 3bl, third bleaching. MM, molecular weight markers.

After fractionation on SDS-PAGE, the macromolecules in the ASM and the LS-AIM fractions were characterized using three staining methods: silver nitrate, Stains-all, and Alcian blue staining (Figure 1). All three show that the two ASMs (2bl and 3bl) exhibited the same electrophoretic pattern, and the two AIMs (2bl and 3bl) too. However, between ASMs and AIMS, differences were noticed. With silver, 5–7 successive discrete bands were detected between 17 and 43 kDa, with two major ones at 35 and 45 kDa in the ASMs. In the two AIMs, a single band at 70 kDa was observed. A band at high molecular weight (>170 kDa) was also visible in all extracts. The overall signals were, however, dominated by smeary ‘polydisperse’ macromolecules (Figure 1A). With Stains-all staining, a broad blue to purple signal was present from about 17 kDa to high molecular weights (>170 kDa) in both ASM fractions (Figure 1B), suggesting that these molecules may bind calcium. In contrast, both AIM fractions were mostly stained pink to red, except a band just below the 70 kDa one, which was slightly stained blue. Note the presence of scaling pattern between 17 and 43 kDa in both AIMs; this scaling pattern was not detected with silver. With Alcian blue staining at low pH (1.0), which detects sulfated polysaccharides and glycosaminoglycans, significant discrete signals were visible at 25 and 50 kDa in ASMs and at 70 kDa in AIMs (Figure 1C), in spite of the intense smearing staining in all extracts.

### FT-IR

**FIGURE 2:**
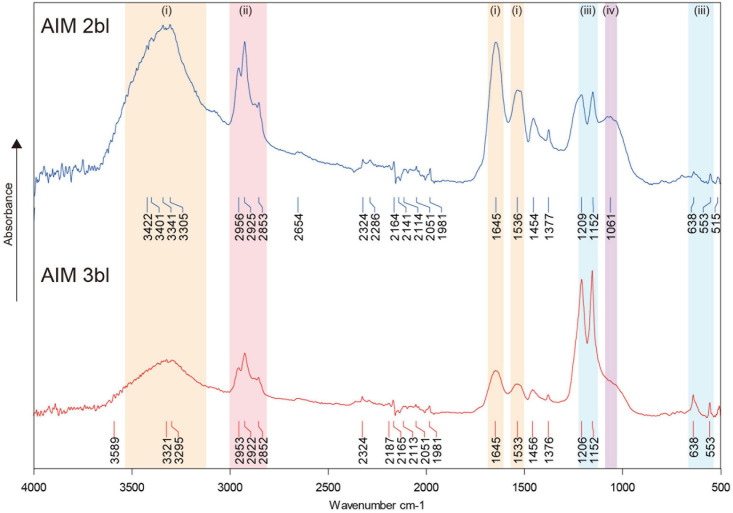
FT-IR spectra of shell organic matrices. The signals in colors indicate the presence of proteins or saccharides (i), lipids (ii), phosphates (iii), and saccharides (iv). AIM, acid-insoluble matrix. 2bl, second bleaching. 3bl, third bleaching.

FT-IR spectra of AIM showed typical absorption bands derived from proteins, lipids, and saccharides (Figure 2). In 2nd bleaching fraction, major signals of a protein backbone were found at around 3,300–3,400, 1,645, and 1,535 cm^–1^, which correspond to amide A (ν_N__–__H_), amide I (ν_C=O_), and amide II (ν_*C*__–__N_) bands, respectively. Absorptions in the range of 2,850–2,950 cm^–1^ corresponding to ν_C__–__H_ stretching vibrations, were also detected and may correspond to lipids. An absorption band specific to carbohydrate was observed near 1,061 cm^–1^ (ν_C__–__O_). In addition, peaks are detected at 1,454–1,456 and 1,376–1,377 cm^–1^, corresponding to adsorptions by carboxylic groups and by ν_C__–__H_ bending, respectively (Marxen et al., 1998). These sharp signals in AIM 2bl were significantly reduced after 3rd bleaching. In contrast, bands located around 550–640 and 1,150–1,200 cm^–1^ can be attributed to characteristic vibrations of phosphate groups, ν_*P*__–__O_ (stretching) and δ_O__–__P__–__O_ (bending) respectively (Jastrzębski et al., 2011; Campos et al., 2021), which were sharply highlighted after 3rd bleaching. This result indicates that the proteins, lipids, and saccharides moieties were reduced by the 3rd bleaching while phosphate groups were retained after this last cleaning step.

### Saccharide Analyses

**FIGURE 3:**
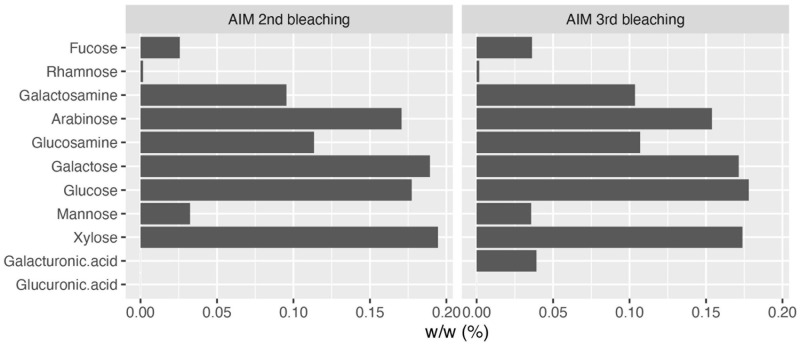
Monosaccharide composition of AIM after two (left) or three (right) bleachings. The third bleaching did not produce any significant change in the relative percentages of monosaccharides in AIM.

**FIGURE 4:**
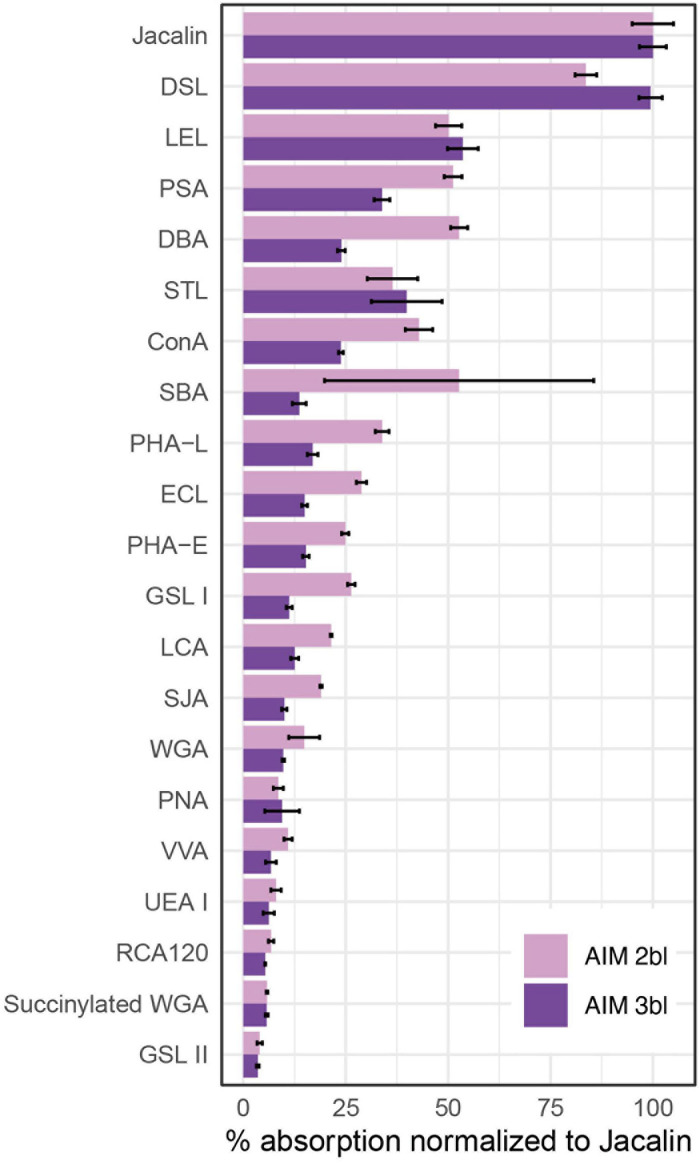
Enzyme-linked lectin assay (ELLA) on ASMs. The test was performed with 21 lectins after two or three bleaching steps. Absorbance values at 405 nm were normalized to the highest value (Jacalin), corresponding to 100% reactivity (*n* = 3, means ± S.D.). Please refer Table 3 of Kanold et al. (2015) for lectin targets. ConA, concanavalin A; DBA, *Dolichos biflorus* agglutinin; DSL, *Datura stramonium* lectin; ECL, *Erythrina crista-galli* lectin; GSL I, *Griffonia simplicifolia* lectin I; GSL II, *Griffonia simplicifolia* lectin II; LCA, *Lens culinaris* agglutinin; LEL, *Lycopersicon esculentum* lectin; PHA-E, *Phaseolus vulgaris* lectin E; PHA-L, *Phaseolus vulgaris* lectin L; PNA, peanut agglutinin; PSA, *Pisum sativum* agglutinin; RCA, *Ricinus communis* agglutinin; SBA, soybean agglutinin; SJA, *Styphnolobium japonicum* agglutinin; STL, *Solanum tuberosum* lectin; UEA I, *Ulex europaeus* agglutinin I; VVA, *Vicia villosa* agglutinin; WGA, wheat germ agglutinin.

Monosaccharide analyses performed on the AIM fractions showed that the relative proportions of each hexose did not vary significantly after 2 or 3rd bleaching steps (Figure 3). The four major monosaccharides in 2bl AIM included xylose (19.4%), galactose (18.9%), glucose (17.7%), and arabinose (12.1%). In the 3bl AIM, each of these monosaccharides represented more than 15% of total amount. Glucosamine and galactosamine showed moderate percentage (around 10%) and mannose and fucose, around 5%. Galacturonic acid was only detected in 3bl fraction. Glucuronic acid was absent from both extracts and rhamnose present in extremely low amounts (<1%). In order to investigate polysaccharidic structure in the ASM fractions, and to obtain their respective lectin-binding signatures, we conducted enzyme-linked lectin assay (ELLA, Figure 4). Both ASM fractions showed the strongest affinities for jacalin, and for *Datura stramonium* lectin (DSL). Jacalin is a α-D galactose-binding lectin that is specific of O-linked oligosaccharides while DSL, which binds oligomers and monomers of *N*-acetylglucosamine, is usually considered as a chitin-binding lectin. Signals of weaker amplitude (between 25 and 50% of that of jacalin, for the two extracts) were obtained with LEL, STL, and PSA. The two first are chitin-binding lectins while the third one binds α-linked mannose-containing oligosaccharides. Additional lectins (DBA, ConA, SBA, PHA-L, ECL, and GSL-1) gave signals higher than 25% for the 2bl extracts but lower than this value for 3bl one. All the other lectins were almost unreactive with the two extracts. Interestingly, DSL was the single lectin for which a significant increase of the relative intensity was obtained after 3rd bleaching; this signal was maintained for LEL and STL but significantly reduced for all other tested lectins, after 3bl treatment.

### Proteomic Analysis

**FIGURE 5:**
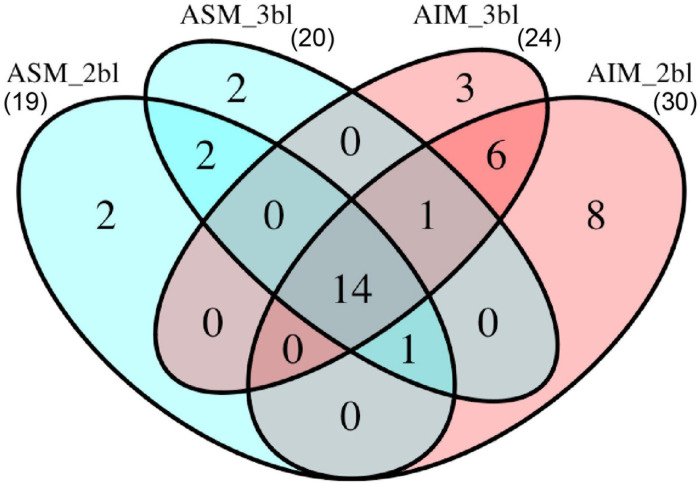
Number of proteins identified from different sample treatments. In total, 39 proteins were identified. ASM, acid-soluble matrix. AIM, acid-insoluble matrix. 2bl, second bleaching. 3bl, third bleaching.

In total, 39 SMPs were identified from the *T. crocea* shells, as shown by the Venn diagram of Figure 5. Fourteen SMPs were common to the four extracts, and two additional SMPs to three of the four extracts. Six SMPs were found solely in ASM extracts (“ASM-specific”) while 17 were “AIM-specific.” We classified these SMPs on the basis of three criteria: the presence of known conserved domains (11 hits), the occurrence of LCRs (19 hits), the absence of these two primary structure characteristics (9 hits). The results are shown in Table 1. Conserved domains were sub-categorized according to their putative functions: affinity to polysaccharides (7 hits), enzymatic activities (2 hits), and protease inhibitors (2 hits). Because some of the identified proteins with conserved domains exhibit modular architecture containing LCRs, we are aware that grouping them in three categories is simplistic and may not reflect the fact that they likely exert different molecular functions in biomineralization.

**TABLE 1:** Classification of *T. crocea* shell matrix proteins.

**Category**	**Protein ID**	**Signal peptide**	***Trans*-membrane**	LCR 1AA*	LCR 2AA*	**Pfam/Prositeprofiles/Superfamily**
Affinity to polysaccharides	Tcr_63362	Yes	No			Chitin binding domain (PF01607) Concanavalin A-like lectin/glucanase domain (SF49899)
	Tcr_356908	Yes	No	T-rich		Chitin binding domain (IPR002557)
	Tcr_684094	Yes	Yes			TSP1 (PF00090) Chitin binding domain (PF01607) VWA (SSF53300)
	Tcr_684124	Yes	No	P-rich T-rich	GQ-rich GS-rich GT-rich	VWA (PF00092) Chitin binding domain (IPR002557)
	Tcr_684209	No	No	G-rich P-rich T-rich	GM-rich GP-rich	Chitin-binding, domain 3 (PF03067)
	Tcr_713741	Yes	Yes			C-type lectin fold (SSF56436)
	Tcr_714405	Yes	Yes	T-rich		Chitin-binding, domain 3 (PF03067)
Enzymes	Tcr_0.575573	Yes	No			Glycoside hydrolase, family 5 (PF00150)
	Tcr_588947	Yes	No			Tyrosinase copper-binding domain (PF00264)
Protease inhibitors	Tcr_531040	Yes	Yes	P-rich		Kazal domain (IPR002350)
	Tcr_824966	No	No			Pancreatic trypsin inhibitor Kunitz domain (PF00014)
Uncharacterized proteins	Tcr_53656	No	No			–
	Tcr_292514	Yes	No			–
	Tcr_311651	Yes	No			–
	Tcr_425966	Yes	No			–
	Tcr_428448	No	No			–
	Tcr_453318	Yes	No			–
	Tcr_526755	Yes	Yes			–
	Tcr_595442	Yes	No			–
	Tcr_652688	Yes	No			–
LCR-containing proteins	Tcr_16185	Yes	Yes	G-rich	GY-rich PQ-rich	–
	Tcr_83017	Yes	Yes	G-rich		–
	Tcr_325918	Yes	No	P-rich	AP-rich	–
	Tcr_366778	Yes	No	K-rich	PT-rich	–
	Tcr_393634	Yes	No	A-rich	AP-rich	–
	Tcr_402398	No	No		AG-rich GS-rich	–
	Tcr_438950	No	Yes	T-rich		–
	Tcr_459507	Yes	Yes	A-rich G-rich P-rich S-rich T-rich	AG-rich GP-rich KV-rich	–
	Tcr_467133	No	No	P-rich T-rich		–
	Tcr_529389	No	No		GM-rich GP-rich GQ-rich	–
	Tcr_564223	Yes	No	Q-rich		–
	Tcr_589629	No	No		GS-rich	–
	Tcr_597367	Yes	No	G-rich K-rich	GM-rich PV-rich	–
	Tcr_618259	No	Yes	G-rich R-rich	GI-rich RS-rich	–
	Tcr_647748	Yes	No	K-rich T-rich	RS-rich	–
	Tcr_654638	No	Yes	D-rich	DK-rich DL-rich DN-rich	–
	Tcr_675074	Yes	Yes	L-rich	GR-rich	–
	Tcr_696598	Yes	Yes	L-rich T-rich		–
	Tcr_714321	Yes	No	GM-rich		–

*Low complexity region composed of one (1AA) or two (2AA) major amino acid(s).

**FIGURE 6:**
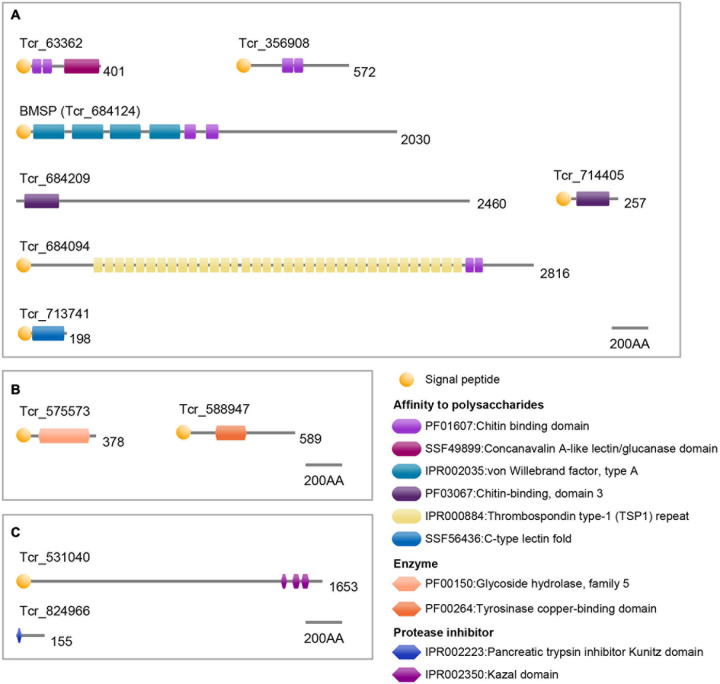
Functional domain architecture of *T. crocea* SMPs. **(A)** Affinity to polysaccharides. **(B)** Enzymes. **(C)** Protease inhibitors. Lengths of amino acid sequences are shown at the right of each protein.

**FIGURE 7:**
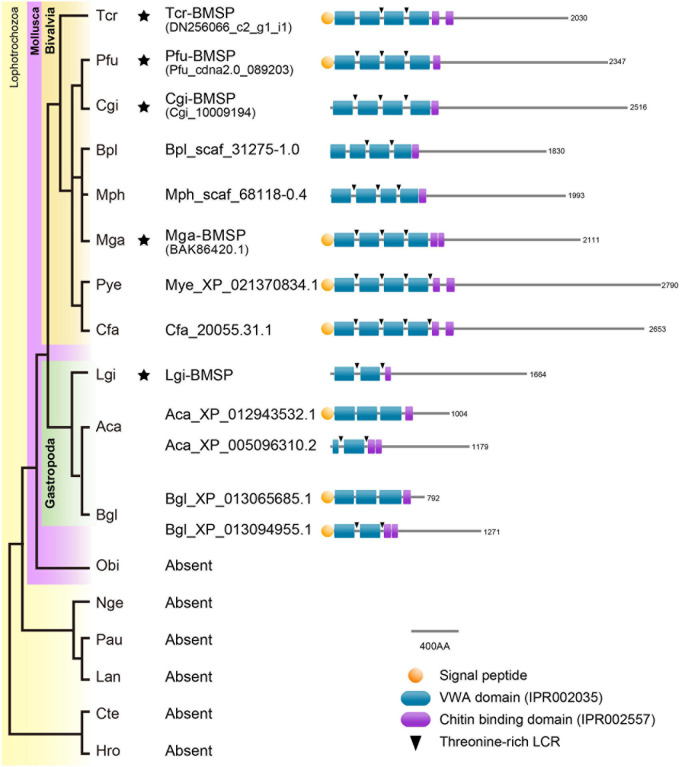
Conserved domain architecture of BMSPs in mollusks. BMSPs carry more than one VWA domain at their N-termini, immediately followed by one or more chitin-binding domains. This domain architecture is only found in molluscan genomes. Asterisks indicate that proteomic analyses confirmed the presence of BMSP in the shells. Species name abbreviation: Aca, *Aplysia californica*, Bgl, *Biomphalaria glabrata*, Bpl, *Bathymodiolus platifrons*, Cfa, *Chlamys farreri*, Cgi, *Crassostrea gigas*, Cte, *Capitella teleta*, Hro, *Helobdella robusta*, Lan, *Lingula anatina*, Lgi, *Lottia gigantea*, Mga, *Mytilus galloprovincialis*, Mph, *Modiolus philippinarum*, Mye, *Mizuhopecten yessoensis*, Nge, *Notospermus geniculatus*, Obi, *Octopus bimaculoides*, Pau, *Phoronis australis*, Pfu, *Pinctada fucata*, Tca, *Tridacna crocea*.

Seven SMPs were characterized by possible affinity to polysaccharides based on functional domain prediction (Figure 6A and Table 1). These SMPs have one or more chitin-binding domain(s) (ChBD) except for Tcr_713741. Among the ChBD-containing proteins, three SMPs including Tcr_63362, Tcr_684124, and Tcr_684094, also carry functional domains such as Concanavalin-A, von Willebrand factor type A (VWA), and Thrombospondin type-1 (TSP-1) domains. These domains are typically found in proteins of the extracellular matrix (ECM). In particular, a SMP Tcr_684124 exhibits four VWA domains followed by ChBD domain(s), showing the characteristic domain architecture of blue mussel shell proteins, i.e., BMSPs identified in pteriomophid bivalves (Figure 7) (Suzuki et al., 2011; Zhao et al., 2018). This is the first report of BMSP ortholog from heterodont bivalves. We consequently name this protein Tcr-BMSP. At last, one SMP (Tcr_713741), which sequence exhibits a calcium-dependent (C-type) lectin fold is also classified in this category (Figure 6A).

Our proteomic search also identified 2 SMPs that have conserved functional domain related to enzymatic activity (Figure 6B and Table 1). The two hits include glycoside hydrolase (Tcr_575573) and tyrosinase copper-binding domains (Tcr_588947). The first one is involved in hydrolyzing glycosidic bonds between two saccharides or between a saccharide and a non-saccharidic moiety. Such domains may be involved in matrix remodeling and reorganization in extracellular environment. The second one, tyrosinase copper-binding domain, catalyzes the hydroxylation of monophenols and the oxidation of *o*-diphenols to *o*-quinols and thus, may be involved either in matrix cross-linking or in shell pigmentation, or both.

At last, two SMPs carried protease inhibitor domains, including trypsin inhibitor Kunitz domain (Tcr_824966) and serine protease inhibitor Kazal domain (Tcr_531040). These domains are known to inhibit the proteolytic activity of a large set of proteases. They are consequently considered as exerting a protective function regarding the shell matrix.

The second category of SMPs comprise 19 proteins characterized by low-complexity regions, abbreviated as LCRs. Hereafter we refer to these proteins as low-complexity region-containing proteins or LCR-CPs. They are all characterized by a significant enrichment of their overall sequence in one or two amino acid residues, as indicated in Table 1. The residues involved in the enrichment can be aliphatic (G, A, V, L, I, and P), basic (K and R), hydroxylated (S and T), acidic (D), amidated (Q), sulfur-containing (M), or aromatic (F). Only four LCR-CPs showed partial sequence similarity to known proteins in the public database: Tcr_393634 to serine protease inhibitor (although this SMP did not have the functional domain), Tcr_589629 to uncharacterized shell matrix protein of *Lottia gigantea* (Table 1). Other two proteins (Tcr_529389 and Tcr_564223) were hit to hypothetical proteins. In addition, LCRs were found in 4 ChBD-containing proteins (Tcr_714405, Tcr_356908, Tcr_684209, and Tcr_684124) and in 2 enzymatic domain-containing SMPs (Tcr_824966 and Tcr_531040) (Table 1 and Supplementary Table 1).

Among the LCR-CPs, the most common are those with hydrophobic aliphatic amino (Table 1), since they represent 16 hits out of 19. Paired hydrophobic amino acids residues compose LCR in Tcr_325918 (AP-rich), Tcr_402398 (AG-rich), Tcr_393634 (AP-rich), and Tcr_459507 (GP-rich). Acidic LCR is found in one hit only, Tcr_654638, having 2–15 consecutive aspartic acids in its C-terminus (Table 1 and Supplementary Table 1). Its theoretical isoelectric point is 3.44, which classifies this SMP as very acidic. Note that one G- and R-rich protein (Tcr_618259) has a poly-Asp(5) sequence in the C-terminus (Supplementary Table 2), although this pattern did not fit to criteria for LCR in this study. Basic LCRs composed of lysin or arginine residues are present in 4 proteins (Tcr_618259, Tcr_366778, Tcr_597367, and Tcr_647748).

**FIGURE 8:**
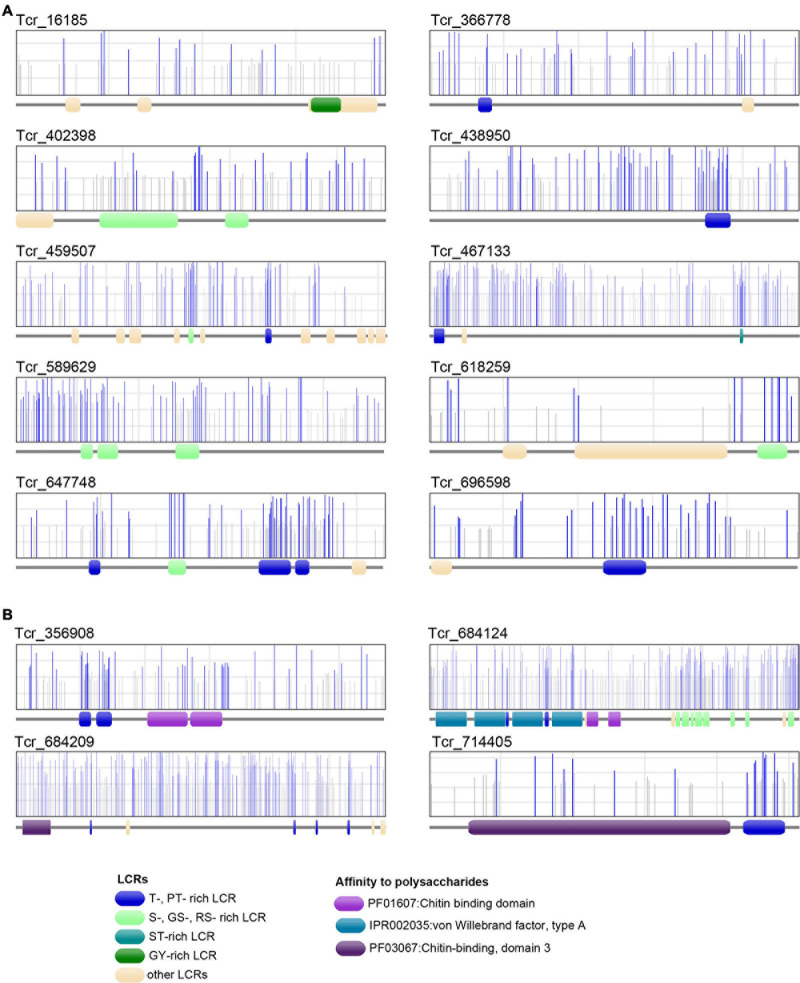
Phosphorylation sites of 10 *T. crocea* SMPs. Each protein primary structure corresponds to the gray horizontal trait with figurations symbolizing conserved functional domains (LCRs, VWA, and ChBDs) dispersed along the sequence. In the rectangles above, vertical lines (gray or blue) represent the positions of putative phosphorylation sites along the sequences, as predicted by NetPhos 3.1. Blue bars indicate likely phosphorylation sites (probability > 0.5), and gray lines, unlikely phosphorylation sites. **(A)** Six LCR-containing proteins. **(B)** Four Chitin-binding domain-containing proteins. Note that the protein regions with the highest density of likely phosphorylation sites often correspond to T-, MT-, PT-, S-, GS-, ST-rich LCRs.

Low-complexity regions exhibiting several putative phosphorylation sites on serine or threonine residues were found in 6 LCR-CPs (Tcr_696598, Tcr_438950, Tcr_589629, Tcr_459507, Tcr_647748, and Tcr_467133). T-rich LCRs are also present in 4 ChBD-containing proteins (Tcr_714405, Tcr_356908, Tcr_684209, and Tcr_684124). A computational prediction of phosphorylation sites demonstrated that these S- or T-rich LCRs are often phosphorylated (Figure 8). For example, SMP Tcr_459507 exhibited 12 LCRs including a S-rich and a T-rich LCRs, and showed significant phosphorylation probabilities in both the S-rich and T-rich regions (Figure 8). Phosphorylation sites were also likely present on S-rich and T-rich LCRs of ChBD-containing proteins such as BMSP.

At last, the third category of *T. crocea* SMPs comprises 9 hits (23% of the SMPs identified here) that did not show any significant similarities to proteins with known conserved domains. One of them (Tcr_311651) exhibits a high similarity with a hypothetical protein of unknown function from the edible oyster *C. gigas*.

## Discussion

Here we report the first biochemical and molecular characterization of the shell organic constituents of the Western Pacific crocus clam, *T. crocea*. Our analysis confirms the complex nature of this skeletal matrix, which contains proteins, polysaccharides and lipids; this latter fraction, detected only via FT-IR spectroscopy, was not investigated further. Our discussion focuses, first on the saccharide moiety, secondly on the protein one.

### Saccharide Moiety

Both AIMs exhibit peculiar monosaccharide composition, with xylose, galactose, glucose and arabinose as the main residues. Note that xylose is not frequently represented in such proportions in matrices associated to CaCO3 skeletons (Cuif et al., 1996). The case of glucosamine should be emphasized: the detection of this residue can result from the hydrolysis of glucosamine-containing polysaccharides or can ensue from the deacetylation of *N*-acetyl-glucosamine - the monomer of chitin – during hydrolysis. Thus, glucosamine in skeletal matrices is often interpreted as a marker of chitin. In our AIM characterization, glucosamine, although relatively abundant, is not dominant, suggesting that the saccharide moiety consists of a mixture of chitin and other types of saccharides of unknown primary structure. This finding is confirmed by ELLA test on ASM where jacalin, the most reactive lectin, indicates the prominence of D-galactose or of *O*-linked oligosaccharides. DSL, the second most reactive lectin, marks the presence of monomers/oligomers of *N*-acetylglucosamine, suggesting that soluble derived products of chitin are present in the ASM. We suppose that the release of such soluble components ensue from the partial hydrolysis and solubilization of chitin by bleach, similarly to what occurs in a chitin-rich biomineral (Oudot et al., 2020). This also suggests that chitin - accessible to bleach – is *intercrystalline*.

The presence of chitin in shells has been reported among several pteriomorph ‘nacro-prismatic’ bivalves: histochemical studies detected chitin in the prismatic layer of the pearl oyster *P. fucata* (Suzuki et al., 2007), in the nacreous layer of the winged oyster *Pteria hirundo* (Osuna-Mascaró et al., 2015), and in the larval shell of the Mediterranean mussel *M. galloprovincialis* (Weiss and Schönitzer, 2006). A recent study found chitin and its derivative, chitosan, in the crossed lamellar shell layer of two heterodont bivalves, the thin-ribbed cockle *Fulvia tenuicostata* and the giant clam *T. gigas*, a closely-related species to *T. crocea* (Agbaje et al., 2019). Our results obtained by two different techniques (HPAE-PAD and ELLA) confirms this latter finding: the presence of chitin in a ‘non-pteriomorph,’ crossed-lamellar bivalve model, where it is believed to participate – via the formation of chitin-protein complexes - to the 3D-structuring of the organic framework. Recent data (Agbaje et al., 2018; Oudot et al., 2020) show that chitin, which importance in shell formation is commonly admitted, is not universally distributed in this exoskeleton. Understanding its exact role in mineral deposition, analyzing its distribution and abundance across the Mollusca and detecting which polymer replaced it functionally in chitin-less shells are major evolutionary questions that deserve attention.

### Proteins of the Shell Matrix

Our proteomic data give a snapshot of the protein composition of the crocus clam’s shell matrix. Thirty-nine proteins were identified, they divide in three groups: those with one or more well identified domain (like enzymatic domains, protease inhibitors or domains interacting with chitin), those with Low Complexity Regions (LCRs), and finally, uncharacterized proteins, i.e., proteins that cannot be affiliated to any of these two categories. Our data give a glimpse on the macroevolution of shell mineralizing matrices in a poorly investigated bivalve clade.

### Chitin and Chitin-Binding Proteins

While sugar analyses strongly suggests the presence of chitin in the matrix of *T. crocea*, another major clue is the proteomic identification of six shell proteins that contain chitin-binding domains (ChBDs). Formerly, several proteome analyses detected several ChBD-containing proteins in bivalve shells (Arivalagan et al., 2017; Marie et al., 2017; Zhao et al., 2018). Despite their commonality, their evolutionary relationships are not clarified because their sequence similarity is restricted to the ChBD, and their overall primary structures are highly divergent, due to their modular architectures. Interestingly, we have identified, in the *T. crocea* shell proteome, an ortholog of BMSP, a ChBD-containing protein originally identified in the blue mussel shell (Suzuki et al., 2011). The BMSP family members are clearly distinguishable from other ChBD-containing proteins from their primary structure, by exhibiting four N-terminal VWA domains in tandem followed by one or two ChBDs (Suzuki et al., 2011). Genes encoding proteins with such domain architecture (Figure 7) are exclusively found in bivalve genomes (Zhao et al., 2018). In contrast, gastropod genomes have one or more putative BMSP gene homologs that encode proteins with only two or three VWA domains followed by one/two ChBDs (Zhao et al., 2018). Many SMPs, in particular those with LCDs or RLCDs, show rapid molecular evolutionary rate (McDougall et al., 2013; Kocot et al., 2016) and putative extensive domain shuffling (Marin et al., 2007). The finding of *T. crocea* BMSP shows that the architecture of the four tandem N-terminal VWA domains was established before the split between Pteriomorphia and Heterodonta sub-classes, an event that dates back to 500 Mya (Plazzi and Passamonti, 2010). The intact conserved domain architecture of BMSPs in two phylogenetically distant bivalve clades indicates that these secreted proteins play likely essential role in shell formation, and that strong functional constraints have maintained the domain architecture of bivalve BMSPs across the Phanerozoic eon. In summary, besides fast-evolving SMPs, ‘shellomes’ also contain SMPs that are remarkably evolutionarily conserved.

In addition to SMPs with ChBDs, LCR-containing proteins may also be involved in binding chitin, such as the GY-rich LCR of Prismalin-14, identified in the calcitic prismatic layer of *P. fucata* (Suzuki et al., 2004; Suzuki and Nagasawa, 2007). In the *T. crocea* shell proteome, Tcr_16185 contains a GY-rich LCR in its C-terminus that may display similar function. Interestingly, GY-rich sequences that putatively bind chitin were also found in pearl oyster SMPs like MSI31 (Sudo et al., 1997), shematrins (Yano et al., 2006), or KRMPs (Liang et al., 2015). So far, the chitin-binding ability of these SMPs has not been tested yet *in vitro*.

### Enzymes and Protease Inhibitors

Tyrosinases represent a family of copper-containing enzymes that oxidize phenol groups of tyrosine into *o*-quinones to induce cross-links. They are responsible for melanogenesis in diverse organisms. They are incorporated as SMPs in the prismatic and nacreous layers on *P. fucata* shells (Nagai et al., 2007; Liu et al., 2015) and nacre of unionoid shells (Marie et al., 2017). Tyrosinases are also identified from different bivalve shell microstructures, including that of three pteriomorphid and one heterodont species (Arivalagan et al., 2017). The presence of tyrosinase in the crossed lamellar shell of *T. crocea* (Figure 6) underlines the widespread utilization of this protein family for shell formation in bivalves, where it works in hardening the organic matrix (cross-linking), in innate immunity, in wound healing and not solely in shell pigmentation (Nagai et al., 2007). Tyrosinases are probably part of an ancient toolkit in mollusk evolution, recruited early for shell mineralization: it was shown that this gene family has undergone complex evolutionary history, with multiple independent evolutions and gene expansion, inactivation or loss in different bivalve lineages (Aguilera et al., 2014).

Protease inhibitors become a ‘recurrent theme’ in mollusc shell proteomes as proteins with protease inhibitor domains have been detected in several shell matrices (Marie et al., 2012; Arivalagan et al., 2017). In *T. crocea* SMPs, we identified two protease inhibitors, a trypsin inhibitor with a Kunitz domain (Tcr_824966) and a serine protease inhibitor with Kazal domains (Tcr_531040). It is generally believed that such proteins are part of a protective mechanism of the calcifying matrix during its secretion, by preventing its premature degradation by proteolytic enzymes in the extracellular environment. We cannot exclude that such domains perform additional unsuspected functions in the context of shell mineralization.

### LCR-Containing Proteins

Despite the prevalence of LCRs in SMPs, the function of most of them in shell formation is still elusive. Among the compositionally biased amino acids in LCRs, hydrophobic aliphatic amino acids (alanine and glycine) are common, as shown by Table 1. Gly-rich or Ala-rich domains are usually considered to exert ‘structural’ (cement between crystal units) or ‘mechanical’ (enhancer of fracture toughness) functions. Because of their hydrophobicity, they may also play a completely different role by expelling water molecules from the system, catalyzing the conversion of amorphous transient water-rich minerals to a crystalline stable form, aragonite. Proline-rich domains are also to be noted because they provide rigid rod-like structures (such as in mucins), but their functional significance is unknown. Basic domains (lysine-rich and arginine-rich) are found in 4 SMPs and their functions give rise to different hypotheses. Two families of basic domain-containing shell proteins have been identified in pteriomorphid bivalves, KRMPs (Liang et al., 2015) and shematrins (Yano et al., 2006). In KRMPs, the basic domains are suspected to inhibit the precipitation of calcium carbonate, to interact with calcite and modify its morphology and to inhibit the growth of aragonite (Liang et al., 2015). We may suggest additional roles: interaction with bicarbonate ions, anchoring of polyanionic polymers via electrostatic interactions. However, these putative functions need to be tested *in vitro*. At last, Table 1 lists a couple of proteins with glutamine-rich domains. Proteins with similar property have been detected in the shell matrix of the gastropod *Haliotis asinina* (Marie et al., 2010) and it has been proposed that they might be involved in protein aggregation. Here again, this hypothesis requires *in vitro* experimental evidences.

### Acidic Proteins

Acidic proteins, i.e., polyanionic proteins in physiological pH conditions, are key components of calcification process: they are indeed supposed to be involved in nucleation, inhibition, and orientation of crystal growth (Addadi and Weiner, 1985; Albeck et al., 1993; Marin and Luquet, 2008). In particular, Asp-/Glu-rich or poly-Asp/poly-Glu domains – a special case of LCRs – are usually considered as regions that bind high amount of Ca^2+^ ions with a moderate affinity, via the negatively charged side chains of Asp/Glu residues (Hare, 1963; Weiner and Hood, 1975; Takeuchi et al., 2008; Suzuki et al., 2009). Bulk amino acid composition demonstrated that Asp was significantly enriched in bivalve shell matrices (Weiner and Hood, 1975; Weiner, 1979, 1983). However, to date, few acidic SMPs with high proportion of acidic AA residues (Asp + Glu >20%) and low theoretical pI (<3.5) have been identified from pteriomorph bivalves: they include MSP-1 and MSP-2 of the scallop *Patinopecten yessoensis* (Sarashina and Endo, 1998, 2001; Hasegawa and Uchiyama, 2005), Aspein of the Japanese pearl oyster *P. fucata* (Tsukamoto et al., 2004; Isowa et al., 2012) and a collection of isoforms, the Asprich family of the rigid pen shell *Atrina rigida* (Gotliv et al., 2005). The shell matrix of *T. crocea* conforms, to a certain extent, to the concepts outlined above on acidic shell proteins. Firstly, it exhibits polyanionic properties, as shown by Alcian blue staining (ASM + AIM) and has the likely ability to bind calcium ions, as indicated by Stains-all (ASM, only). Secondly, we have identified the full sequence of a novel acidic protein (Tcr_654638, pI = 3.44, Asp + Glu = 32.4%) with a D-rich LCR in its C-terminus (Table 1 and Supplementary Table 2), which does not show any significant sequence similarity to known proteins in public databases. This suggests its independent origin from pteriomorph D-rich proteins (MSP-1, Aspein) and possibly, a clade-specific recruitment within heterodont bivalves.

In addition to the presence of acidic residues, phosphorylation may contribute to the acidic nature of some SMPs (Marin and Luquet, 2007). Phosphate groups are detected in matrices associated to calcium carbonate biominerals of diverse metazoan animals such as the sea urchin *Arbacia lixula* (Kanold et al., 2015), the zebra mussel *Dreissena polymorpha* (Immel et al., 2016), or the scleractinian coral *Porites australiensis* (Takeuchi et al., 2018). Our FT-IR result showed that phosphate groups are retained in the matrices (AIMs) after intense bleach, suggesting their strong affinity with the biomineral (Figure 2).

Phosphorylated proteins, in particular those with Ser-rich/Thr-rich LCRs, are found in various biominerals. In vertebrates, they include osteopontin, a bone matrix protein (Gericke et al., 2005) but also dentin matrix protein (DMP-1) and phosphophoryn (aka DMP2) two highly phosphorylated teeth proteins (George et al., 1993; He et al., 2005). Phosphorylation is crucial for both proper folding and calcium binding ability of phosphophoryn (He et al., 2005). Among non-vertebrate calcium carbonate biominerals, Orchestin (Hecker et al., 2003) and CAP-1 (Inoue et al., 2003) are phosphorylated proteins involved in calcium storage during ecdysis in crustaceans, and their calcium-binding ability depends on phosphorylation. Phosphorylated acidic proteins (phosphodontin) were found in the teeth system (Aristotle’s lantern) of the sea urchin *Strongylocentrotus purpuratus* (Mann et al., 2010).

In the *T. crocea* shell proteome, 10 SMPs have S- or T- rich LCRs that are likely phosphorylated (Figure 8). Four ChBD-containing proteins including BMSP exhibit putative phosphorylated LCRs, which certainly increase their affinity to calcium ions. We suggest that the combination of ChBDs and T-rich LCRs in some SMP sequences may be an essential requisite to form a molecular bridge between chitin and calcium carbonate. Phosphorylation may also change the conformation of SMPs to build proper structure in the shell organic framework. Our results highlight the phosphorylation of SMPs as an alternative mechanism to ‘acidify’ the shell organic matrix, regardless of the presence of Asp-rich proteins.

## Conclusion

In this study, we conducted a biochemical characterization of the crossed lamellar shell of *T. crocea*, the crocus clam. We identified 39 proteins that show little homology with that of other bivalves studied so far. Beside evolutionary aspects, the SMP repertoire of *T. crocea* provides a large set of molecular markers, usable to check how this species reacts to environmental stress, in particular ocean acidification, known to induce deleterious effects on shell calcification. We have every reason to think that these effects may be quantifiable by analyzing the SMP gene expression.

At last, the SMP repertoire of living *T. crocea* opens a window on the shell repertoire of similar fossil shell materials. Recently, we have identified SMPs from well-dated (2,880 ± 30 BC) *Tridacna* sp. subfossils of French Polynesia (Marin et al., 2018). Our study shows the good potential for preservation of some SMPs across archaeological times. It consequently paves the road of an emerging discipline, ‘palaeoshellomics’ (Wallace and Schiffbauer, 2016; Sakalauskaite et al., 2020).

## Data Availability Statement

The data presented in the study are deposited in the Dryad repository, accession number https://doi.org/10.5061/dryad.4mw6m9094.

## Author Contributions

TT and FM conceived and designed the experiments. TT, MF, RK, LP, IZ-D, NB, and CB performed the experiments and analyzed the data. TT, NS, and FM wrote the manuscript. All authors reviewed and approved the manuscript.

## Conflict of Interest

The authors declare that the research was conducted in the absence of any commercial or financial relationships that could be construed as a potential conflict of interest.
